# Demographic and Habitual Factors of Periodontal Disease among South Indian Adults

**DOI:** 10.3390/ijerph18157910

**Published:** 2021-07-26

**Authors:** Siddharthan Selvaraj, Nyi Nyi Naing, Nadiah Wan-Arfah, Mauro Henrique Nogueira Guimarães de Abreu

**Affiliations:** 1 Faculty of Medicine, Medical Campus, Universiti Sultan Zainal Abidin, Kuala Terengganu, Terengganu 20400, Malaysia; sidzcristiano@gmail.com; 2 Faculty of Health Sciences, Universiti Sultan Zainal Abidin, Kuala Terengganu, Terengganu 20400, Malaysia; wanwaj@unisza.edu.my; 3 School of Dentistry, Universidade Federal de Minas Gerais, Belo Horizonte 31270-901, Brazil; maurohenriqueabreu@gmail.com

**Keywords:** periodontal disease, epidemiology, habits, logistic model

## Abstract

The aim of this study was to evaluate the performance of a set of sociodemographic and habits measures on estimating periodontal disease among south Indian adults. This cross-sectional study was carried out among 288 individuals above 18 years old in Tamil Nadu, India. The outcome of the study was periodontal disease, measured by WHO criteria. The covariates were age, ethnicity, smoking and alcohol habit. The assessment of factors predicting periodontal disease was carried out by multiple logistic regression analysis using R version 3.6.1. The demographic factors like age group (AOR = 3.56; 95% CI 1.69–7.85), ethnicity (AOR = 6.07; 95% CI 2.27–18.37), non-alcoholic (AOR = 0.31; 95% CI 0.13–0.64) and non-smoking (AOR = 0.33; 95% CI 0.15–0.67) were found to be associated with the outcome. The maximum log likelihood estimate value was −30.5 and AIC was 385 for the final model, respectively. Receiver operating characteristic (ROC) curve for the periodontal disease was 0.737. We can conclude that sociodemographic factors and habits were useful for predicting periodontal diseases.

## 1. Introduction

Oral diseases are considered to have a wide presence around the globe, which is associated with remarkable morbidity. The Indian population in particular have a considerable imbalance towards their oral health care [1]. Considering oral conditions among Indian adults, about 25% of adults experience mild to moderate periodontitis, on the other hand severe periodontitis is seen among 19% adults [2]. Periodontal disease is an inflammatory chronic condition that damages the tissues that surrounds teeth [3] and can also be considered a global public health problem, as it is rising in every region among all socioeconomic classes [4]. The steady rise in periodontal disease can be stalled by investigating the peridontium of an individual at the early stage of periodontitis, resulting in better oral health-related quality of life. Periodontium can be investigarted with the help of Community Periodontal Index (CPI). World Health Organization indicates CPI to investigate the oral health status of the community population and to screen periodontal status. CPI has limitations, such as extensively estimating the severity of periodontal condition by giving the highest score without considering the clinical attachment loss [2]. Despite its limitations, CPI is still widely used in oral health surveys [5] and this is the most commonly utilized index to measure and estimate the prevelence of periodontal diseases. Moreover, there is a lack of consensus to provide a benchmark on the diagnostic measurement of periodontal disease [6]. Despite the difference in measuring periodontal disease in an epidemiological setting, this condition has an impact on an individual’s self-esteem, causing halitosis, and impacting the quality of life [7,8,9]. Moreover, there are good evidences of the relationship between periodontal disease and other systemic conditions [9,10]. In the year 2000, the surgeon general of the United States emphasized that wellness of oral health is crucial for general health wellbeing [11]. It has been concluded by experts that periodontitis causes bacterial burden, which results in a systemic inflammatory response, and is also a contributing factor of the pathophysiology of various systemic diseases, such as cardiovascular diseases, diabetes, and metabolic syndrome [12]. In addition, a WHO board of executives have identified the relationship between general health, quality of life and oral health [13]. Sociodemographic factors are one of the risk factors of periodontal disease and various oral conditions. The disparity of oral hygiene habits among individuals from different socioeconomic backgrounds results in poor oral hygiene status. Families with low incomes show poor oral hygiene maintenance of their child. The income and education of an individual has a relationship with periodontal status. Individuals who have a low income have a higher probability of getting periodontal disease than individuals with a high-income [14]. It has been well documented that ethnicity also plays a major role in periodontal conditions. An individual, study carried out by Borrell and colleagues in the year 2006 found prevalence of periodontal conditions was higher among African Americans than Hispanic and non-Hispanic white Americans [15]. Likewise, it has been found that the prevalence of periodontal disease is lower when an individual’s level of education is higher, thus the education of an individual influences their periodontal status. In addition, the presence of periodontal disease can be related to a person’s lifestyle. If an individual follows a healthy lifestyle, they have less chance of getting periodontal disease when compared to an individual who follows an unhealthy lifestyle. It has been found that there is a consistent relationship with behaviour and socioeconomic factors of an individual with periodontal conditions [16]. The assessment of periodontal disease in an Indian population was not performed as regularly as dental caries. Hence, there is still a lack of information on the epidemiology of periodontal disease among the Indian population [2]. A study by Francis and his colleagues in the year 2017, found there was a well-established association between demographic characteristics and periodontal status. Sociodemographic and socioeconomic characteristics play a major role in the periodontal health of an individual [17], however there is a lack of data that justifies the association of periodontal disease with demographic characteristics and a person’s individual habits. Hence, the aim of this study was to analyse the association of sociodemographic factors, habits, and periodontal disease among Indian adults.

## 2. Materials and Methods

A cross-sectional study was carried out among South Indian adults who reside in Tamil Nadu.

### 2.1. Study Participants, Sample Size Determination and Sampling Method

Study participants were residents of Chennai-Tamil Nadu. Adults who live in a residential community located in Chennai-Tamil Nadu and who satisfied the inclusion criteria were considered to be a part in this study. Participants above 18 years of age who volunteered to take part in our research were considered in this study. The contents of the questionnaire were in English. Hence, individuals who were capable of reading and understanding English took part in our study. English is one of the official languages in India and the second most commonly spoken language [18]. The literacy rate in the study location—Chennai—is 90.18% [19], thus English language will not be a barrier to our study [20]. To support the above data, majority of the participants in this study were well educated and have attained a good educational qualification. This shows that there will not be any bias regarding language criteria. Edentulous individuals who did not have a single tooth were not considered in this study. The sample size of our study was planned based on the recommendation by Palmer [21] for calculating the disparities on periodontal disease among adults. Although we met Palmer’s criterion on sampling, we performed a posterior power calculation for some covariates. The statistical power for the sample size was 64.34%. A total of 288 adults who participated were selected using the systematic random sampling method. Study samples were selected in a fixed periodic interval. The sampling interval was determined by splitting the overall population size by the required sample size. Systematic sampling was utilized when the participants are logically homogeneous, since systematic sample units are distributed in a uniform manner. In the sampling frame, the beginning point is selected randomly, and choices subsequently are at periodic intervals.

### 2.2. Ethics Approvals and Data Collection Procedures

Ethics approvals were obtained from the Human Research Ethics Committee of Universiti Sultan Zainal Abidin and RIPON independent ethics committee affiliated with the Government of India, before carrying out this study. Before questionnaire distribution, an informed consent was obtained from the individuals who volunteered themselves to be a part of our study. In addition, study purpose, study procedure, and study confidentiality were explained to the individuals who were willing to participate in the study.

Data collection for this study took place in the month of February 2021. The participants were asked to answer a validated questionnaire which is presented in Table 1. To validate our questionnaire, we carried out an exploratory factor analysis in our previous study and contents in our questionnaire had satisfactory factor loading. Furthermore, confirmatory factor analysis of our questionnaire had ideal goodness of fit values and had good convergent and discriminant validity. The results of our confirmatory factor analysis had statistically significant values [1]. The questionnaire took nearly 5 to 10 min to complete. An investigation on periodontal profile was carried by following SOP, provided by the state health department as dentists are more prone to being infected by 2019-nCoV, due to the specificity of the infection [22]. Dental examination was carried out by a unique trained dental public health specialist, who had received ideal knowledge on periodontal disease from an expert in the field of oral epidemiology for one year, which was a part of the curriculum during his speciality training. The primary investigator examined periodontium of the participants under artificial light by utilising disposable gloves, a CPI probe, and a dental explorer. All the instruments used during our study were sterilized [23]. Examination of periodontal profile by CPI index were noted based on the diagnostic criteria recommendation given by WHO [24]. A CPI probe was utilized to investigate six sites of every tooth that includes mid buccal, distobuccal, mesio-buccal, mesio-lingual, distolingual and mid-lingual to investigate bleeding on probing (BOP) and probing pocket depth (PPD). PPD denotes gingival sulcus depth and denotes periodontal disease status (Table 2) [25]. A survey that was carried out to investigate the periodontal status among the south Indian population stated that, out of 1075 participants, 537 individuals had dental calculus. Out of these 537 participants, bleeding of gums with gingivitis was seen among 76 individuals. So, calculus and gingivitis are quite associated and taken into account as a periodontal condition [26]. We dichotomized the outcome in absence of any periodontal disease (CPI = 0) and the presence of any other conditions (CPI > 0) [27].

### 2.3. Statistical Analyses

In a logistic model, we contemplate the response as random categorical variables, obtaining only two probable results, known as dichotomous or binary results. Data obtained in our study were analysed using R version 3.6.1. Dichotomized data of periodontal disease were analysed. A descriptive analysis of categorical variables were carried out. 

Data was cleaned and explored to check missing and duplicate data. A simple logistic regression and multiple logistic regression model was developed among binary periodontal disease response variables, alongside independent factors (Table 3). Simple logistic regression was carried out to determine potential candidate varaibles for multivariable analysis. Principles of best fit, biological plausibility and statistical significance, with a *p*-value of less than 0.25, were applied to obtain variables to include in multiple logistic regression analysis. As suggested by Hosmer and his colleagues in the year 2013, we used *p* < 0.25 to select potential covariates to be included in the final model. However, only covariate *p*, 0.05 were retained in this final logistic regression model [28]. Stepwise forward and backward procedures were carred out to obtain a parsimonious model, which was considered to be the preliminary main effect model. Multicollinearity was checked by using variance inflation factor (VIF) and correlation matrix. VIF less than 10 was considered to be free of multicollinearity problems. Biologically meaningful two-way interaction terms were checked by using a likelihood ratio (LR) test to obtain the preliminary final model. The fit of the model was checked by applying the Hosmer–Lemeshow Goodness of Fit test, overall correctly classifed percentage, and area under Receiver Operating Characteristic (ROC) curve to obtain the final model. 

To weigh the significance level of selected variables based on their influence, its association with model, and the assessment of adjusted odds ratios (OR), 95% confidence intervals and corresponding *p*-values of individual factors were determined [27]. Log likelihood ratio of the variables were measured to establish goodness of the fit model, in which the higher the value the better the model. In addition, Akaike Information Criterion (AIC) was also measured to estimate the likelihood of the model to predict the subsequent values. Minimal AIC score indicate a better model fit. 

## 3. Results

A total of 288 individuals were included in the current study, from which 121 (42%) had periodontal disease (CPI > 0). The majority of participants (*n* = 147; 51%) were male. The distribution of participants among age groups of 18–24 years, 25–34 years, 35–44 years and ≥ 45 years were found to be 68 (23.6%), 65 (22.6%), 84 (29.1%) and 71 (24.7%), respectively. Other descriptive information can be found on Table 4.

Out of 13 factors, only four factors (age, ethnicity, smoking habit and alcohol habit) were found to be statistically significant factors of a periodontal profile (*p* < 0.05), with a log likelihood value of −30.457 and Akaike’s information criterion (AIC) value of 384.96. Table 4 presented the logistic regression models that were developed to predict the periodontal disease of patients with 13 independent factors. The estimated crude and adjusted OR (95% CI) and *p*-values of the final model were presented on Table 5. 

Individuals aged 25–34 years and more than 45 years were found to have 3.5 times (AOR = 3.56; 95% CI 1.69–7.85) and nearly 2.5 times (AOR = 2.37; 95% CI 5.21–11.10) higher odds of having periodontal disease, respectively, when compared to 18-24 years age group. In participants with an ethnicity other than Tamil, the odds of them haing periodontal disease were six times higher (AOR = 6.07; 95% CI 2.27–18.37) when compared to participants with Tamil ethnicity. The odds of having periodontal disease was found to be 69% less among individuals who did not consume alcohol (AOR = 0.31; 95% CI 0.13–0.64), when compared to the participants who consumed alcohol. Nonsmokers were 67% less likely to have periodontal disease (AOR = 0.33; 95% CI 0.15–0.67) than smokers.

## 4. Discussion

Periodontal disease is one of the common and major diseases which can lead to chronic inflammation, the destruction of tooth supporting structures, and tooth loss in adults [29], though as it is chronic condition it can be treated, even at the initial stage by non-invasive treatments, utilizing diode lasers which have excellent treatment outcomes [30]. In addition, dental implants can also be considered as an adequate clinical option for rehabilitation of patients who lost their tooth due to periodontal conditions [31,32,33].

Developing a model would be an ideal option to predict the factors of periodontal disease. There are various statistical methods, such as logistic regression, multilevel modelling, and ordinal regression methods, can be utilized to analyse periodontal disease. By utilizing regression analysis, it is possible to know the associated factors of various factors such as demography and other related factors that affect the oral health status of the respondents. These techniques allow us to study the magnitude of the factor effect. Hence, the data of periodontal disease were modified into binary outcomes or dichotomous outcomes i.e., with periodontal disease and without periodontal disease. To the best of our knowledge, this study is the first one to assess the association of sociodemographic factors and periodontal disease among south Indian adults. The general aim of the present study was to predict the association of periodontal diseases with socio-demographic and habitual factors. The prevalence of periodontal disease observed in the current study was 42%. From our study, it was revealed that the incident of periodontal disease among 288 participants was highly dependent on the age, ethnicity, alcohol consumption, and smoking status of the individuals. 

Based on our study result age groups of 25–34 years and ≥45 years had shown higher prevalence of the disease. The prevalence and severity of periodontal disease tends to increase with age of patients [34]. It is also stated that there are differences in the prevalence of periodontal disease in different age groups [35]. Identical findings were reported in a study which explained that the severity of periodontal disease aggravates due to absence of treatment, resulting in disease progression with the aging of an individual [36]. Periodontal disease severity and range were found to be divergent in different age groups and the common trend perceived in the major number of the studies showed expanded periodontal severity with increase in age [37]. It can be said that it is not the age of a person that is responsible for high prevalence of periodontal disease, but relatively the duration of periodontal tissue, which is confronted with the accumulation of chronic plaque in an individual’s periodontium [38]. Based on the study findings, it can be said that age can be one of the influencing factors of periodontal disease [39]. 

Based on the study results by Shen and his colleagues in the year 2013, socioeconomic factors of an individual, such as income, employment, education, and residence, have an influence on the oral health of an individual, which shows that these factors affect inequalities in oral health status [40]. The income of an individual might play a role in an individual’s ability to access oral health care services, which may affect the outcome of their oral health. Education plays a role due to the lack of utilization of oral health care services, due to the lack of interaction with service providers and lack of use of oral health information received [41]. Considerable ethnic differences in periodontal disease between and within different ethnicities have been reported [42]. It has been well-related with our study that there is an association between socioeconomic factors and periodontal disease, and it also correlates with the outcomes of various studies [40]. Recent investigations showed that consuming a high amount of alcohol might alter the host defence mechanisms of an individual, and it is well-established that intake of alcohol is linked with high prevalence of infections and might have effect on an individual’s periodontium [43]. 

The above findings were in line with this study, in which the prevalence of periodontal disease was lower among individuals who did not consume alcohol, when compared to the individuals who did consume alcohol. Studies have found that periodontal diseases are associated with individuals who smoke, and smoking is a well-established factor causing periodontal diseases [44]. In addition, it can be well established that the smoking habits of an individual is one of the strongest risk factors associated with the development and progression of periodontal disease [45]. The level of Cotinine serum between 10 and 20 ng/mL was found to be compatible with smoking individuals and resulted in high severity of periodontal disease [46] and correlates well to our study, in which periodontal disease was more common among the participants who smoked, compared to non-smoking individuals. 

Our present study has few limitations. As our study is a cross-sectional study, it is not possible for us to establish causal direction of periodontal disease and it only provides the relationship of periodontal disease with the outcomes that have been analysed. It is known that periodontal disease has a well-established association with systemic diseases, diabetes mellitus, metabolic syndrome, and cardiovascular disease. An assessment on the association of periodontal disease with systemic conditions was excluded, as it was beyond the scope of our study. As a result, it can be seen as one of the important limitations of our study [12]. Contextual variables were not able to be justified, due to participants’ change of address. Furthermore, the edentulous individuals who had a possible link with periodontal conditions were also excluded from our study. There are feasible associations between demographic factors and habits and oral health inequality which need to be explored vastly. Nevertheless, providing better solutions for treatment facilities provides better wellbeing for an individual. Based on the outcome of this study, exploring and providing information regarding the risks and behaviours of periodontal disease at timely intervals helps to achieve a better upliftment of oral health among individuals, helping them to plan a better oral health policy alongside the concerned authorities. 

## 5. Conclusions

From our study, we found that the age, ethnicity, smoking and alcohol habits of an individual were considered to be notable periodontal disease associated factors among south Indian adults. Our findings shed light on how various sociodemographic factors and the role of an individual’s personal habits determines periodontal health. Furthermore, this study explains the inequalities regarding oral health among individuals and assists in the development of an ideal policy on oral health care, which will help to provide a better insight into the factors of periodontal disease and remove inequalities so that individuals can maintain better oral hygiene.

## Figures and Tables

**Table 1:** Oral health KAB Questionnaire.

Knowledge	Attitude	Behaviour
1. There are two sets of teeth during lifetime	1. Brushing teeth twice a day improves oral hygiene	1. I give importance to my teeth as much as any part of my body
2. Tooth infection causes gum bleeding	2. Keeping your teeth clean and healthy is beneficial to your health	2. I have sensitive teeth
3. Replacement of missing tooth improves oral hygiene	3. Improper brushing leads to gum disease	3. I brush my tooth twice daily
4. The dental caries of deciduous teeth need not be treated	4. Sweet’s retention leads to tooth decay	4. I use teeth to open cap of bottled drink
5. Bacteria is one of the reasons to cause gingival problems	5. Brushing with fluoridated toothpaste prevent tooth decay	5. I experience tooth ache while chewing food
6. Fizzy soft drinks affect the teeth adversely	6. Dentists care only about treatment & not prevention	6. I have bleeding gums during brushing
7. Loss of teeth can interfere with speech	7. Gum bleeding denotes gum infection	7. I do routine dental check-up
8. Irregularly placed teeth can be moved into correct position by dental treatment	8. Scaling is harmful for gums	
9. Decayed teeth can affect the appearance of a person		
10. Tobacco chewing, or smoking can cause oral cancer		
11. White patches on teeth are called dental plaque		

**Table 2:** Scoring criteria of periodontal disease based on WHO criteria.

Score	Interpretation
0	Healthy gums with absence of periodontal disease
1	Bleeding on probing
2	Calculus felt during probing
3	Presence of periodontal pocket with depth between 3.5 mm and 5.5 mm
4	Presence of periodontal pocket with depth 6 mm or more

**Table 3:** Associated factors analysed among adults, Tamil Nadu, India, 2021.

Variable	Coding
Gender	1. Male 2. Female
Age	1. 18–24 2. 25–34 3. 35–44 4. ≥45
Marital status	1. Married 2. Single
Religion	1. Hindu 2. Muslim 3. Christian 4. Others
Ethnicity	1. Tamil 2. Others
Diet	1. Vegetarian 2. Non-Vegetarian 3. Mixed
Smoking	1. Yes 2. No
Alcohol consumption	1. Yes 2. No
Education	1. Illiterate 2. Primary 3. High school 4. University
Employment status	1. Employed 2. Unemployed 3. Student 4. Homemaker
Income *	1. Below 10 k ₹ 2. 10 k ₹–20 k ₹ 3. 20 k ₹–30 k ₹ 4. Above 30 k ₹
House ownership	1. Owned 2. Rented
Own Vehicle	1. Yes 2. No

* 10 k ₹ equals to $137.76 American Dollars in February 2021.

**Table 4:** Sociodemographic and habits profile of the adult population (*n* = 288), Tamil Nadu, India, 2021.

Variable	Categories	*n*	%
Gender	Male Female	147 141	51.0% 49.0%
Age	18–24 25–34 35–44 ≥45	68 65 84 71	23.6% 22.6% 29.1% 24.7%
Marital status	Married Single	192 96	66.7% 33.3%
Religion	Hindu Muslim Christian Others	237 15 30 6	82.2% 5.2% 10.6% 2.0%
Ethnicity	Tamil Others	264 24	91.6% 8.4%
Diet	Vegetarian Non-Vegetarian Mixed	32 52 204	11.1% 18.2% 70.7%
Smoking	Yes No	40 248	13.9% 86.1%
Alcohol consumption	Yes No	35 253	12.2% 87.8%
Education	Illiterate Primary High school University	7 12 93 176	2.6% 4.7% 31.6% 61.1%
Employment status	Employed Unemployed Student Homemaker	186 23 46 33	64.6% 8% 16% 11.4%
Income	Below 10 k ₹ 10 k ₹–20 k ₹ 20 k ₹–30 k ₹ Above 30 k ₹	98 50 72 68	34.1% 17.3% 25% 23.6%
House ownership	Owned Rented	178 110	61.8% 38.2%
Own vehicle	Yes No	180 108	62.5% 37.5%

**Table 5:** Demographic and habitual factors of periodontal disease among adult population in Tamil Nadu, India, 2021 by using simple and multiple logistic regression analyses (*n* = 288).

Variable	Simple Logistic Regression			Multiple Logistic Regression ^a^		
	Regression Coefficient (b)	Crude Odds Ratio (95% CI)	*p*-Value	Regression Coefficient (b)	Adjusted Odds Ratio (95% CI)	*p*-Value
Ethnicity						
Tamil	0	1		0	1	
Other than Tamil	1.55	4.69 (1.90–13.29)	0.002	1.8	6.07 (2.27–18.37)	<0.001
Age						
18–24 years	0	1		0	1	
25–34 years	1.09	2.98 (1.47–6.18)	0.003	1.28	3.56 (1.69–7.85)	0.001
35–44 years	0.29	1.33 (0.67–2.67)	0.41	0.26	1.30 (0.63–2.73)	0.480
≥45 years	0.85	2.33 (1.17–4.75)	0.02	0.86	2.37 (5.21–11.10)	0.029
Smoking						
Smoked	0	1		0	1	
Did not smoke	−0.97	0.38 (0.19–0.75)	0.006	−1.12	0.33 (0.15–0.67)	0.003
Alcohol						
Consumed	0	1		0	1	
Did not consume	−0.83	0.44 (0.21–0.89)	0.02	−0.98	0.31 (0.13–0.64)	0.002

^a^ Forward Stepwise Likelihood Ratio Multiple Logistic Regression model was applied. Multicollinearity and interactions were checked and not detected. Hosmer–Lemeshow Goodness of fit test (*p* = 0.9136) and area under ROC curve (71.4%) were applied to check the fit of the model.

## Data Availability

The datasets used in this study are obtainable from the corresponding author on request.

## Abbreviations

MLR	Multiple Logistic Regression
SLR	Simple Logistic Regression
ROC	Receiver Operating Characteristic
WHO	World Health Organization
CPI	Community Periodontal Index
PPD	Probing Pocket Depth
BOP	Bleeding on Probing
VIF	Variance Inflation Factor
